# Histological and molecular characterisation of feline humeral condylar osteoarthritis

**DOI:** 10.1186/1746-6148-9-110

**Published:** 2013-06-04

**Authors:** John M Ryan, B Duncan X Lascelles, Javier Benito, Jon Hash, Sionagh H Smith, David Bennett, David J Argyle, Dylan N Clements

**Affiliations:** 1 Royal (Dick) School of Veterinary Studies and Roslin Institute, Division of Veterinary Clinical Sciences, Hospital for Small Animals, Easter Bush Veterinary Centre, The University of Edinburgh, Roslin, Midlothian EH25 9RG, Scotland; 2 Department of Clinical Sciences, Comparative Pain Research Laboratory & Center for Comparative Medicine and Translational Research. College of Veterinary Medicine, North Carolina State University, 1060 William Moore Drive, Raleigh, NC 27606, USA; 3 The School of Veterinary Medicine, College of Medical, Veterinary and Life Sciences, University of Glasgow, Bearsden Road, Bearsden, Glasgow G61 1QH, Scotland

**Keywords:** Osteoarthritis, Feline, Humeral condyle, Gene expression, Histomorphometry

## Abstract

**Background:**

Osteoarthritis (OA) is a clinically important and common disease of older cats. The pathological changes and molecular mechanisms which underpin the disease have yet to be described. In this study we evaluated selected histological and transcriptomic measures in the articular cartilage and subchondral bone (SCB) of the humeral condyle of cats with or without OA.

**Results:**

The histomorphometric changes in humeral condyle were concentrated in the medial aspect of the condyle. Cats with OA had a reduction in articular chondrocyte density, an increase in the histopathological score of the articular cartilage and a decrease in the SCB porosity of the medial part of the humeral condyle. An increase in LUM gene expression was observed in OA cartilage from the medial part of the humeral condyle.

**Conclusions:**

Histopathological changes identified in OA of the feline humeral condyle appear to primarily affect the medial aspect of the joint. Histological changes suggest that SCB is involved in the OA process in cats. Differentiating which changes represent OA rather than the aging process, or the effects of obesity and or bodyweight requires further investigation.

## Background

Osteoarthritis (OA) is a pathological change of a diarthrodial articulation characterised by the deterioration of articular cartilage, osteophytosis, bone remodelling, mineralisation in the periarticular tissues and a low grade non-purulent inflammation [1]. Although OA is often used synonymously with the term degenerative joint disease (DJD), OA is a disease only of synovial joints whereas DJD covers pathology of non-synovial joints such as spondylosis and degenerative lesions of joints which are not part of OA, such as traumatic enthesiopathies. Awareness of OA and DJD in the feline population has increased in recent years [2] and a high radiographic prevalence of OA and DJD has been observed in general cat populations [3-5]. The prevalence of OA and DJD increase with age in cats [6,7], mirroring the pattern of distribution of OA in human populations [8]. Osteoarthritis commonly affects the elbow joint of cats [1,9].

Histological changes in the cartilage of joints with OA have been reported in many mammalian species. The development of clefts, the loss of proteoglycan, changes in cellularity and the loss of tidemark integrity of articular cartilage of OA joints are features which characterise the disease [10] and which can be used to grade its severity. These features are not species specific, thus grading schemes such as the Mankin Histological and Histochemical Grading System (HHGS) [10] have been used to record the severity of the disease in many different species. Recognition of concurrent changes in other articular tissues such as synovium [11] and subchondral bone (SCB) [12,13] has led to the widespread appreciation that OA is a disease which affects all the tissues in a joint.

The molecular changes characterising OA include the differential expression of matrix proteins, proteoglycans, collagens, metalloproteinases and their inhibitors in both cartilage and bone. The patterns of identified molecular changes are dependent on the species [14], joint [15], location within the joint [16] and stage of the disease process [17,18] although broad similarities in patterns of differential gene expression in end-stage OA are observed between species [14].

The development of SCB pathology in OA is believed to have a critical role in the pathogenesis and progression of the disease [19,20]. The thickness of the SCB plate [21] and density of trabeculae in the SCB increases in naturally occurring OA [22]. The temporal relationship between the development of cartilage and SCB pathology in naturally occurring OA is unclear, and has not been well defined in experimental models of the disease. Changes in SCB have been recorded at the same time point as cartilage pathology [23,24], or they may be identified after the development of cartilage pathology [25,26].

As the histological, and transcriptomic features of feline OA have not been correlated with gross or radiographic features, previously, the aim of this study was to report a preliminary description of the histological features and transcriptomic changes in the articular cartilage and SCB of the humeral condyle in populations of cats with or without naturally occurring OA. Our hypothesis was that there would be histological or molecular differences between cats with or without gross or radiographic OA change.

## Results

Cats without elbow OA were younger (mean 3.92 years (SD 1.0) versus mean 10.33 (1.4), P = 0.002), weighed significantly less (mean 3.08 kg (0.36) versus 6.03 (0.65), P = 0.011) and had a significantly lower body condition score (median 4/9, versus 6/9, P = 0.0149) than the OA group. The median radiographic score for OA and normal joints were 1 (range 0 – 4) and 0 (range 0–0) respectively. The median gross pathological score for the lateral and medial aspects of OA joints was 0 (range 0 – 2) and 3 (range 2–4), respectively. Signalment details are presented in Additional file 1.

When each joint was assessed individually, articular cartilage was significantly thicker at the central part of the humeral condyle in OA joints (245.3 μm (14.0)) compared to the normal joints (203.1 (11.0), P = 0.027). No differences were observed in the articular cartilage thickness at the medial (OA joints 212.1 (13.0), normal joints 194.2 (12.0), P = 0.582) or lateral parts of the joint (OA joints 203.5 (13.0), normal joints 194.5 (8.9), P = 0.175). When the average value for each cat was evaluated, no significant differences were observed at the medial (P = 0.758), central (P = 0.091) or lateral (P = 0.318) parts of the condyle, between OA and normal cats.

The mean chondrocyte density in the articular cartilage of the medial part of the humeral condyle was significantly reduced in the OA joints (780 cells/mm^2^ (55)) when compared to normal joints (1099 (98), P = 0.009). No differences were identified in the mean chondrocyte density at the lateral (OA joints 922 (132), normal joints 1050 (102), P = 0.510) and central (OA joints 764 (64), normal joints 830 (67), P = 0.434) parts of the humeral condyle. When the average value for each cat was evaluated, chondrocyte density in the articular cartilage of the medial part of the condyle remained significantly reduced in OA cats, compared to normal cats (P = 0.003). No differences were evident on the central (P = 0.754) or lateral (P = 0.336) parts of the condyle between OA and normal cats.

The median OARSI-COH grade in the medial part of the humeral condyle in the OA joints was significantly higher (2.0, IQR 1.125-3.0, P = 0.008) than that of the normal joints (0.0, 1–1.0). No differences were identified in the median OARSI-COH grade at the lateral (OA cats 1.0, 0.125-2.0; normal joints 0.0, 0–1.0, P = 0.146) or central (OA cats 1.25, 0–2.0; normal cats 0, 1.0-1.5, P = 0.332) parts of the humeral condyle. When the average value for each cat was evaluated, median OARSI-COH grade remained significantly increased on the medial part of the humeral condyle on OA cats, compared to normal cats (P = 0.008). No differences were evident on the central (P = 0.332) or lateral (P = 0.17) parts of the condyle between OA and normal cats The median HHGS score was significantly higher in the medial part of the humeral condyle of OA joints (5.5. IQR 3.375-7.0) compared to normal joints (1.0, 0.5-2.0, P = 0.002). No differences were observed in the HHGS scores at the lateral (OA joints 2.0, 1.5-2.75; normal joints 1.0, 0.5-2.0, P = 0.231) or central (OA joints 2.5, 0.625-3.75; normal joints 1.0, 0–2.0, P =0.224) parts of the humeral condyle. When the average value for each cat was evaluated, median HHGS score was significantly higher in the medial part of the humeral condyle in OA cats (P = 0.0073). No differences were apparent on the central (P = 0.0938) or lateral (P = 0.0933) parts of the humeral condyle, between normal and OA cats. Strong positive correlations were noted between HHGS score and OARSI-COH grade in the lateral (ρ = 0.798), central (ρ = 0.861) and medial (ρ = 0.896) parts of the humeral condyle.

Mean subchondral osteocyte density (N.Ot/B.Ar) was significantly reduced in the lateral (P = 0.001), central (P = 0.0001) and medial (P = 0.012) parts of the humeral condyle in OA joints (219.4 cells/μm^2^ (23), 270.6 (26) and 291.6 (24), compared to normal joints (368.0 (34), 457.0 (38) and 422.0 (42)). When the average value for each cat was assessed, subchondral osteocyte density was significantly reduced on the central (P = 0.002) and lateral (P = 0.005) parts, but not on the medial (P = 0.102) part of the humeral condyle. SCB porosity (Vd.Ar/T.Ar.) was significantly reduced in the medial part of the humeral condyle in OA joints (7.8 (6.47)) compared to normal joints (15.4 (11.9), P = 0.035). No significant differences in Vd.Ar./T.Ar. were apparent on the lateral (OA joints 9.3% (11.1), normal joints 14.3 (10.4), P = 0.212) or central parts of the condyle (OA joints 3.62 (3.12), normal joints 8.0 (10.3), P = 0.132). When the average value for each cat was evaluated, SCB porosity was significantly reduced in the medial part of the joint (P = 0.035) but not on the central (P = 0.06) or lateral (P = 0.169) parts of the humeral condyle.

**Figure 1:**
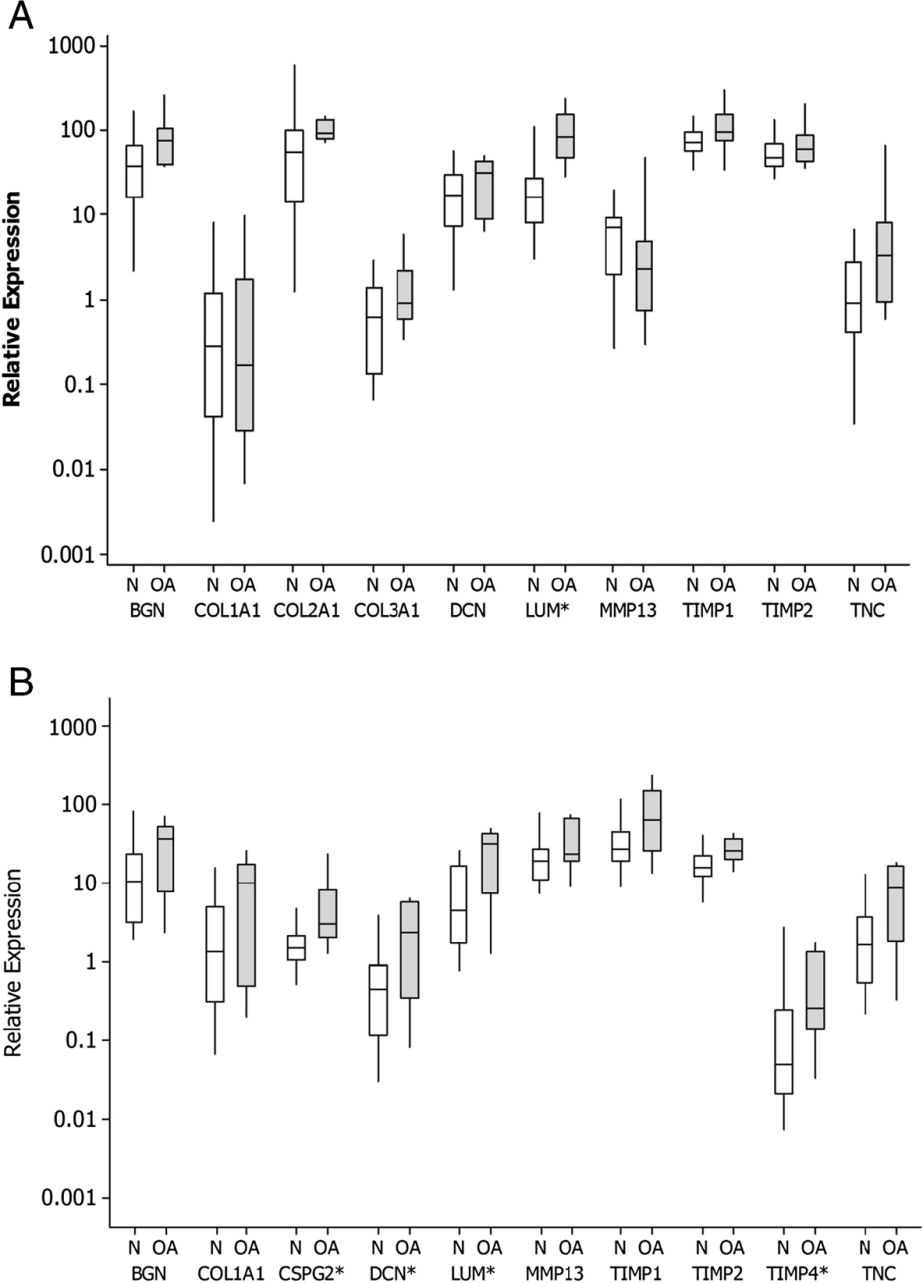
**Median (and interquartile ranges) relative gene expression profiles select genes in articular cartilage (A) and SCB (B) from normal cats and cats with osteoarthritis (OA).** *Significant difference.

The results of the gene expression profiles are illustrated in Figure 1A and 1B, and Additional file 2. In OA cartilage from the medial part of the humeral condyle, a five-fold increase in expression of LUM (P = 0.002) was identified when compared to normal cartilage when each joint was evaluated individually. When the average expression from each cat was evaluated, significant increases in expression of LUM (P = 0.002), was apparent. Significant increases in the expression of CSPG2 (P = 0.048)*,* DCN (P = 0.042), LUM (P = 0.036)*,* and TIMP4 (P = 0.045) were identified in OA SCB from the medial part of the humeral condyle compared to the SCB of medial humeral condyle of the normal specimens, when joints were assessed individually. When the average expression from each cat was evaluated, no significant differences in expression were apparent.

## Discussion

Previous reports suggest that the feline elbow joint commonly develops OA with greater radiographic and macroscopic changes than are observed in other feline appendicular joints [9]. The gross pathological changes are reported to be focused on the medial aspect of the joint at the articulation of the medial coronoid process with the medial part of the humeral condyle [27], which concurs with the histopathological changes found in the humeral condyle in this preliminary study. The microscopic pathological changes in articular cartilage, as assessed by the OARSI and HHGS grading schemes were limited in the population evaluated. End-stage pathologies such as a break in tidemark integrity and clefts to the calcified zone were not noted in any of the tissues evaluated. This reflects that the screened and studied population was not selected on the basis of their clinical presentation, and thus the OA tissues did not include samples from joints which had progressed to end stage disease. Had the cohort consisted of older cats, reaching their natural end of life, more severe histopathological changes may have been expected. Alternatively, the findings may reflect inherent difference in OA in felidae compared to other species.

The thickness of mammalian articular cartilage is broadly related to species size and weight [28,29]; consequently it is unsurprising that feline cartilage is relatively thin. Both articular cartilage thickening and a reduction in cellularity of articular cartilage have been reported in experimental feline OA [30]. The increase in articular cartilage thickness identified at the central part of the humeral condyle in OA joints was unexpected, as the other histomorphological changes in the OA cartilage were primarily observed in the medial aspect of the joint. However these findings were not observed when the average values of each cat were compared, and thus their significance is uncertain.

The positive correlation seen between HHGS and OARSI grades on the lateral, central and medial parts of the humeral condyle was anticipated, and consistent with a previous evaluation of these scoring systems in man [31]. As the OARSI score is comprised of a greater number of elements, it was used with the hope of increasing the sensitivity of detection of subtle articular cartilage lesions, compared to the HHGS alone, in light of speculation regarding the adequacy of this system [32]. The severity of the cartilage pathology, as measured by either grading scheme was only significantly different on the medial aspect of the joint and was not particularly marked, suggesting a relatively early stage of disease. Alternatively, it may be the grading systems used were not appropriate for feline cartilage. Pathology in the canine elbow joint is also almost universally observed on the medial side of the joint [21], possibly as the result of abnormal loading or incongruency [33]. In turn this has led to the development of treatment strategies for end-stage disease which redistribute load through the healthier part of the joint [32]. No gross incongruency of the feline elbow was observed in the present study although incongruency was not specifically assessed, other than by gross visual and radiographic inspection *post-mortem*.

The osteocyte density in SCB was decreased across the humeral condyle when comparing the OA joints, and in the central and lateral parts of the humeral condyle when comparing OA cats. A decrease in osteocyte density has been reported in dogs with fragmentation of the medial coronoid process (MCP) [34], and a decline in osteocyte density in the MCP also has negative correlations with the severity of cartilage pathology and radiographic OA score in dogs [35]. A reduction in osteocyte density has also been noted in femoral necks of human patients with coxofemoral OA, and is hypothesised to arise as a result of increased matrix levels of TGFβ in the OA cartilage [36]. However, increasing age also reduced the osteocyte density in human cancellous bone [37]. Thus, without recourse to evaluation of age and weight matched groups we cannot definitively conclude whether the changes we identified are the result of the aging process or weight differences, which may explain their distribution across the humeral condyle, or whether they are part of, or the result of, the OA disease process.

The reduction in bone porosity (Vd.Ar./T.Ar.) noted in the medial part of the condyle was thought to be part of the OA process, although this contradicts experimental studies which commonly reveal an increase in subchondral porosity [38]. Whether this reflects a temporary, early-stage event [39], or a species difference is unclear from this limited study.

A greater number of genes demonstrated differential expression in this study in subchondral bone compared to articular cartilage when analysed at the level of the joint, but not at the level of the individual cat. Lumican expression was increased in feline elbow OA cartilage, as has been reported in canine elbow OA where its expression correlates with the radiographic severity of the disease [40]. Lumican expression was also increased in SCB. Lumican is believed to have a role in the organisation of collagen fibrils of connective tissues. While knockout studies do not demonstrate a pivotal role for LUM in isolation in bone or cartilage development [41], it does impede the deposition of normal COL2 fibrils in tissue engineered cartilage [41]. Interaction between COL2 and LUM can diminish the ability of MMP13 to cleave COL2 [42] and so impede collagenolysis, thus the increased expression of LUM observed in cartilage may be a protective mechanism to prevent the degradative process. Differential expression of the remaining candidate genes profiled was not observed in feline OA cartilage, possibly reflecting the fact that these genes were selected on the basis of their previously reported expression in end- rather than early-stage OA [14,18,40]. Comparison between these two disease states in human cartilage revealed that little differential expression of the external matrix proteins is observed in the early stage of the disease [18].

Since an extensive analysis was carried out on each humeral condyle, we were limited to relatively small tissue samples for each part of the study. Whilst the anatomical position was consistent between different samples for the histological and molecular measures, they did not necessarily encompass the site of the worst pathology. Only a single histological section was examined which may have resulted in a failure to recognise differences in adjacent tissue. At the time of data acquisition, no validated radiographic evaluation of osteoarthritis in cats was available. A modified IEWG score was used, however, the validity of this score in cats is not known. Joints without both gross and radiographic evidence of OA were excluded to reduce the potential for false assignment of joints. Statistically significant differences in age, body weight and body condition score were found between the normal and diseased populations. The impact of these differences on the variables we assessed is unknown. However, due to the correlation between age and DJD in cats, the high prevalence of OA in older cat populations means it would be very challenging to recruit meaningful numbers of geriatric cats without OA. Body weight and condition score are associated with the development of OA in the canine population [43], and indeed the same may be true in cats. We did not comprehensively evaluate every joint in these cats, and variations in DJD-burden amongst the cats may have influenced the data. Clearly our populations were highly stratified on the basis of their inclusion criteria, and thus further population studies of OA in cats are required to analyse these variables in more detail.

## Conclusion

Based on our findings, histological changes in feline elbow OA appear to be concentrated on the medial part of the humeral condyle. Pathology is seen in the SCB, in the absence of histological pathology in the overlying articular cartilage which is similar to naturally occurring OA of other species. The changes in histological parameters and gene expression were identified in cartilage and SCB, and thus future therapeutic strategies should consider both tissues in the treatment process.

## Methods

The study was approved by the Veterinary Ethical Review Committee of the University of Edinburgh. Twenty five adult cats, euthanatized for reasons unrelated to this study (population control), were evaluated. The consent for use in the study was provided by cat owners (Wake County Shelter and Durham County Shelter). The weight (kg), body condition score (Purina Scoring System [1-9]) [44], breed, age (years), gender and neuter status were recorded. The aim was to recruit cats with radiographically normal elbows and elbows with OA. Cats were considered normal if they had no radiographic evidence of OA and no gross evidence of cartilage damage on the humeral condyle of either elbow joint. Cats were considered to have OA if they had gross evidence of OA on the humeral condyle of each elbow joint and radiographic evidence of OA in either elbow joint.

### Radiographic evaluation

Immediately *post-mortem*, orthogonal radiographs of the cadaveric elbow joints were made, using an indirect digital flat panel imaging system (Canon Medical CXDI-50G Sensor, Eklin Medical Systems, Santa Clara, CA, USA), evaluated using Dell Ultrasharp monitors (2407WFP, Dell, Round Rock, TX, USA) and viewed using image viewing software (eFilm 2.1.2, Merge Healthcare, Milwaukee, WI, USA). Radiographic evaluation of OA was graded using a modified International Elbow Working Group (IEWG) scale, not previously validated in cats (0 = No changes, 1 = osteophytes <2 mm, 2 = osteophytes greater than 2 mm but less than 5 mm, 3 = osteophytes >5 mm) [45]. All radiographs were viewed independently, and under blinded conditions, by JB and BDXL, and then a consensus reached during a combined blinded reading.

### Gross evaluation

Elbow joints were disarticulated for gross observation. The medial and lateral articular surfaces of the humeral condyle (trochlea and capitulum) were examined and scored for gross evidence of cartilage damage (0 = no cartilage pathology, 1 = chondromalacia, 2 = partial thickness fibrillation, 3 = full thickness fissuring, 4 = full thickness erosion, or ulceration) [46]. Based on these scores, each elbow was assigned an OA status. The normal group comprised those with no radiographic evidence of DJD or OA and no evidence of cartilage damage grossly visible in either elbow (n = 11 cats; 22 elbows); the OA group was comprised of cats with radiographic evidence of OA and a positive gross evaluation score (n = 9 cats; 17 elbows; one elbow had gross but no radiographic evidence of OA so was not evaluated); five additional animals (10 elbows) were excluded because they had evidence of cartilage damage on visual inspection in one or both elbows, but no radiographic changes in either joint.

### Tissue samples

The humeral condyle, comprising the medial trochlea and lateral capitulum, was collected from each elbow. The humeral condyle was removed from the humeral diaphysis with an osteotome. The humeral condyle was then osteotomised into two pieces in the mediolateral plane along the long axis of the humerus. The cranial part was stored in a RNA stabilising reagent (RNAlater®, Ambion Inc, Austin, TX, USA) at room temperature for 24 hours and subsequently at −20°C. The caudal part of the humeral condyle was stored in 10% neutral buffered formalin at room temperature.

### Histopathological evaluation

Formalin stored humeral condyles were decalcified using a commercial decalcifier (Surgipath® Decalcifier Leica Microsystems Ltd., Milton Keynes, UK) prior to paraffin embedding and sectioning at 5 μm thickness. Sections were cut perpendicular to the weight bearing surface of the humeral condyle. Serial sections were mounted onto glass slides and stained with haematoxylin and eosin and Safranin-O. The sectioned humeral condyles were evaluated microscopically (Olympus CX21, Lapu Lapu City, Cebu, Philippines) on two separate occasions by a single investigator (JR), blinded to the disease status.

Each section was evaluated using the Mankin Histological and Histochemical Grading System (HHGS) [10]. Articular cartilage was also evaluated using the Osteoarthritis Research Society International (OARSI) Cartilage OA Histopathology grading system (OARSI-COH grade) [47]. HHSG scores and cartilage grades were recorded at the medial (trochlear), central and lateral (capitular) parts of the articular surface of the condyle. The central point was considered to be the part of the articular surface of the humeral condyle with greatest concavity. Images of each sample were digitally captured at 200× magnification and analysed using an image processing and analysis software package (ImageProPlus, Media Cybernetics, Bethesda, MD, USA). A mean cartilage thickness and chondrocyte density (number of chondrocytes per articular cartilage area) was measured above the tide mark at the same points on the medial, central and lateral articular surfaces of each condyle. Osteocyte density (number of osteocyte nuclei per bone area, N.Ot/B.Ar) was measured at points immediately subjacent to the SCB plate and expressed as number of nuclei per mm^2^[48]. Bone porosity (void of bone area (Vd.Ar) in μm^2^ per total bone area (T.Ar)) was calculated by outlining bony trabeculae with a digital marker, which determined the void of bone in μm^2^ and this was expressed as a percentage of the total bone area (T.Ar.) in that section (Vd.Ar. × 100/T.Ar.) [49].

### RNA extraction

Humeral condylar tissue was defrosted and removed from RNAlater®. Articular cartilage was harvested from the medial part of the humeral condyle by sharp scalpel dissection, SCB tissues were harvested from the medial part of the humeral condyle using bone rongeurs. Total RNA was extracted using phenol/guanidine HCl reagents (TRIzol® Reagent, Invitrogen Ltd, Paisley, UK) and isolated as previously described [50] including an on-column DNA digestion step (Qiagen®, RNase-Free DNase Set; Qiagen Ltd, Crawley, UK). Final elution of the total RNA was performed using 30 μl of RNase-free water and repeated to maximize the amount of RNA eluted. Total RNA concentration was quantified using a spectrophotometer (NanoDrop Technologies, Wilmington, DE, USA). RNA integrity was assessed by evaluating the capillary electrophoresis trace of the sample by using the RNA integrity number RIN algorithm [50].

### Synthesis of cDNA

Each sample was normalised to 20 μg/μl using RNase-free water and the reverse transcription was carried out using 10 μl RNA (200 μg total RNA) with oligo-dT_12-18_ and a reverse transcriptase (Superscript ™III RT, Invitrogen Ltd, Paisley, UK). Subsequently, the cDNA was diluted with 500 μl RNase/DNase-free water and stored at −80°C.

### Quantitative PCR

Target genes were selected from those known to be differentially expressed in articular cartilage and/or SCB in OA in other species [14,18,40]. The genes selected for cartilage were biglycan (BGN), type-1 collagen, alpha-1 chain, (COL1A1), type-2 collagen, alpha-1 chain (COL2A1), type-3 collagen, alpha-1 chain (COL3A1), decorin (DCN), lumican (LUM), matrix metalloproteinase-13 (MMP13), tissue inhibitor of metalloproteinase 1 (TIMP1), tissue inhibitor of metalloproteinase 2 (TIMP2), tissue inhibitor of metalloproteinase 4 (TIMP4) and tenascin C (TNC). The genes selected for bone were BGN, COL1A1*,* versican (also called chondroitin sulphate proteoglycan-2, CSPG2), DCN, LUM, MMP13*,* tissue inhibitor of metalloproteinase 1 (TIMP1), TIMP2, TIMP4 and TNC. Four reference genes were evaluated using a gene stability algorithm [51]; 5-aminoimidazole-4-carboxamide ribonucleotide formyltransferase/IMP cyclohydrolase (ATIC), glceraldehyde-3- phosphate dehydrogenase (GAPDH), mitochondrial ribosomal protein S7 (MRP S7) and mitochondrial ribosomal protein S25 (MRP S25). ATIC and MRP S7 were selected for use in the study. Oligonucleotide primers were synthesised by Eurofins MWG Operon (Ebersberg, Germany). Primer and probe sequences were designed using online design software (Universal Probe Library Assay Design Centre, Roche Diagnostics Ltd,http://www.roche-applied-science.com/sis/rtpcr/upl/ezhome.html), and are listed in Additional file 3.

Assays were performed in duplicate using a plate-based quantitative real-time PCR system (Lightcycler® 480 Roche Diagnostics Ltd., Lewes, UK). Ninety-six well plates were employed, with a 10 μl reaction volume, consisting of 4.7 μl sample cDNA (templates), 5 μl 2× PCR Mastermix, 0.1 μl forward and reverse primers and 0.1 μl probe. All assays were performed in duplicate with additional control samples (H_2_O) for each assay, on each plate. Amplification was carried out and analysed according to a standard protocol (Monocolour hydrolysis probe) with 10 minutes at 50°C, followed by 40 cycles of 95°C for 60 s, and 60°C for 15 s. Real time data was analysed using LightCycler® 480 Basic Software (Roche Diagnostics). Standard curves were generated for each assay to confirm that all assays were generated within acceptable limits (efficiency 93% > × > 107%). Real time variables were analysed by generation of the mean threshold cycle (C_T_) value for each transcript in duplicate. Means were calculated for the two reference genes (ATIC and MRP S7) and were used to calculate the ΔΔC_T_ and the relative amounts of each target gene in articular cartilage and SCB [52]. A MiQE checklist [53] is presented in Additional file 4.

### Data analysis

Normal and non-normal data were expressed as mean and standard deviation (SD) or median values and ranges. For all measures, normality was compared using the Kolmogorov-Smirnov normality test. The age and weight of cats were compared with the calculation of means and Student’s *t*-tests. Cartilage thickness, chondrocyte density, SCB porosity and subchondral osteocyte density were compared using Student’s *t* -tests. The HHGS score and OARSI-COH grades were compared using Mann–Whitney *U* tests. The correlation between HHGS score and OARSI-COH grades was assessed using a Spearman’s rank test. Relative expressions of each gene from articular cartilage and SCB were compared using median values and Mann - Whitney *U* tests. Statistical significance was set at P < 0.05 for all tests. Data were checked for errors due to multiple hypothesis testing, using the Benjamini and Hochberg false discovery rate [54]. Statistical tests were carried out using statistical software (Minitab® 15.1.20.0, Minitab Ltd., Coventry, UK) or an internet-based calculator (http://www.maccery.com/maths/). All tests were performed at the individual joint and individual animal level (where the average value for the measure of both joints was used in the comparison) to check for confounding associated with potential correlation between the two joints of an individual.

## Competing interests

The authors declare that they have no competing interests.

## Authors’ contributions

JR was involved in sample processing, data acquisition and manuscript preparation. BDXL, JB and JH collected tissues and carried out radiographic studies. SHS assisted in tissue processing and data acquisition and reviewed the manuscript. DJA and DB contributed to the study concept and reviewed the manuscript. DNC contributed to the study concept and design, sample processing, data acquisition, statistical analysis, and manuscript preparation. All authors read and approved the final manuscript.

## Supplementary Material

Additional file 1 **Signalment of cats, including OA status based on radiographic and gross features.** BCS = Body Condition Score, OA = osteoarthritis, Y = yes, I = intermediate, N = no, M = male, F = female, FN = female neutered, MN = male neutered, DSH = Domestic Short Hair, DSLH = Domestic Semi Long Hair, DLH = Domestic Long Hair. Click here for file

Additional file 2 The median relative expression of genes evaluated in normal and OA feline cartilage and SCB, interquartile ranges (IQR), fold change in OA tissue relative to normal and corrected P value. Click here for file

Additional file 3 Primer and probe sequences. Click here for file

Additional file 4 MiQE checklist. Click here for file
