# Supplementary material for: Histological and molecular characterisation of feline humeral condylar osteoarthritis
Source: BMC Vet Res. 2013 Jun 4;9:110. doi: 10.1186/1746-6148-9-110 (PMC3681712; doi:10.1186/1746-6148-9-110)
Supplement: Additional file 1 — Signalment of cats, including OA status based on radiographic and gross features. BCS = Body Condition Score, OA = osteoarthritis, Y = yes, I = intermediate, N = no, M = male, F = female, FN = female neutered, MN = male neutered, DSH = Domestic Short Hair, DSLH = Domestic Semi Long Hair, DLH = Domestic Long Hair. [file 1746-6148-9-110-S1.docx]

**Table 1** Signalment of cats, including OA status based on radiographic and gross features. BCS = Body Condition Score, OA = osteoarthritis, Y = yes, I = intermediate, N = no, M = male, F = female, FN = female neutered, MN = male neutered, DSH = Domestic Short Hair, DSLH = Domestic Semi Long Hair, DLH = Domestic Long Hair

| **Case No** | **Age (yr)** | **Weight (Kg)** | **BCS /9** | **Gender** | **Breed** | **OA (Y/I/N)** |
| --- | --- | --- | --- | --- | --- | --- |
| **1** | 2.00 | 2.25 | 3 | F | DSH | N |
| **2** | 6.00 | 5.50 | 5 | M | DSH | N |
| **3** | 2.00 | 5.00 | 4 | M | DSH | N |
| **4** | 0.10 | 2.70 | 3 | F | DSH | N |
| **5** | 10.00 | 10.00 | 8 | M | DSH | Y |
| **6** | 9.00 | 3.50 | 4 | FN | DSH | I |
| **7** | 4.00 | 4.00 | 3 | M | DSH | N |
| **8** | 12.00 | 6.44 | 7 | FN | DSH | Y |
| **9** | 2.00 | 4.40 | 4 | M | DSLH | N |
| **10** | 2.00 | 3.60 | 5 | F | DSH | N |
| **11** | 1.00 | 2.40 | 3 | F | DSLH | N |
| **12** | 6.00 | 4.80 | 4 | MN | DSH | Y |
| **13** | 12.00 | 3.20 | 5 | M | DSLH | N |
| **14** | 5.00 | 4.80 | 6 | MN | DSH | I |
| **15** | 8.00 | 5.20 | 7 | M | DSH | I |
| **16** | 5.00 | 3.10 | 3 | F | DSH | N |
| **17** | 11.00 | 8.00 | 8 | MN | DSH | Y |
| **18** | 8.00 | 6.00 | 7 | FN | DSH | Y |
| **19** | 8.00 | 5.40 | 7 | FN | DSH | Y |
| **20** | 7.00 | 5.60 | 6 | MN | DSH | N |
| **21** | 7.00 | 8.00 | 8 | FN | DSH | I |
| **22** | 8.00 | 6.00 | 5 | FN | DSH | I |
| **23** | 13.00 | 4.50 | 5 | MN | DLH | Y |
| **24** | 19.00 | 3.50 | 4 | MN | DLH | Y |
| **25** | 6.00 | 5.60 | 6 | MN | DLH | Y |
