# Supplementary material for: Histological and molecular characterisation of feline humeral condylar osteoarthritis
Source: BMC Vet Res. 2013 Jun 4;9:110. doi: 10.1186/1746-6148-9-110 (PMC3681712; doi:10.1186/1746-6148-9-110)
Supplement: Additional file 2 — The median relative expression of genes evaluated in normal and OA feline cartilage and SCB, interquartile ranges (IQR), fold change in OA tissue relative to normal and corrected P value. [file 1746-6148-9-110-S2.docx]

**Table 2**

The median relative expression of genes evaluated in normal and OA feline cartilage and SCB, interquartile ranges (IQR), fold change in OA tissue relative to normal and corrected P value

| **Gene** | **Tissue** | **Median Normal** | **Median OA** | **IQR Normal** | **IQR OA** | **Fold Change** | **Corrected P Value** | **Fold Change (Cat Average Comparison)** | **Median Corrected P value** |
| --- | --- | --- | --- | --- | --- | --- | --- | --- | --- |
| ***BGN*** | Cartilage | 36.95 | 75.19 | 49.83 | 65.9 | 2 | 0.129 | 2.361 | 0.098 |
| ***COL1A1*** | Cartilage | 0.28 | 0.17 | 1.18 | 1.76 | 0.6 | 1 | 1.464 | 1.000 |
| ***COL2A1*** | Cartilage | 55.6 | 91.1 | 84.5 | 50.7 | 1.6 | 0.097 | 2.0988 | 0.085 |
| ***COL3A1*** | Cartilage | 0.64 | 0.91 | 1.24 | 1.6 | 1.4 | 0.157 | 1.491 | 0.105 |
| ***DCN*** | Cartilage | 17.37 | 31.78 | 22.5 | 33.44 | 1.8 | 0.465 | 1.377 | 0.625 |
| ***LUM*** | Cartilage | 16.28 | 84.16 | 19.18 | 108.6 | 5.2 | 0.002 | 6.058 | 0.002 |
| ***MMP13*** | Cartilage | 6.92 | 2.31 | 7.34 | 3.99 | 0.3 | 0.175 | 0.318 | 0.254 |
| ***TIMP1*** | Cartilage | 70.6 | 94 | 36.07 | 81.8 | 1.3 | 0.117 | 1.739 | 0.114 |
| ***TIMP2*** | Cartilage | 47.92 | 60.97 | 29.72 | 44.8 | 1.3 | 1 | 1.301 | 0.253 |
| ***TNC*** | Cartilage | 0.93 | 3.38 | 2.34 | 7.06 | 3.6 | 0.133 | 2.669 | 0.093 |
| ***BGN*** | Bone | 10.65 | 37.6 | 20.98 | 44.95 | 3.5 | 0.056 | 4.047 | 0.117 |
| ***COL1A1*** | Bone | 1.37 | 10.3 | 4.75 | 16.91 | 7.5 | 0.055 | 5.074 | 0.105 |
| ***CSPG2*** | Bone | 1.54 | 3.01 | 1.13 | 6.16 | 2 | 0.048 | 2.461 | 0.084 |
| ***DCN*** | Bone | 0.47 | 2.38 | 0.83 | 5.46 | 5.1 | 0.042 | 4.344 | 0.142 |
| ***LUM*** | Bone | 4.64 | 31.72 | 14.98 | 36.4 | 6.8 | 0.036 | 4.899 | 0.071 |
| ***MMP13*** | Bone | 19.63 | 24.25 | 16.51 | 48.71 | 1.2 | 0.169 | 1.277 | 0.189 |
| ***TIMP1*** | Bone | 27.7 | 64 | 26.4 | 127.9 | 2.3 | 0.142 | 2.222 | 0.100 |
| ***TIMP2*** | Bone | 16.11 | 26.4 | 9.77 | 16.11 | 1.6 | 1 | 1.372 | 0.101 |
| ***TIMP4*** | Bone | 0.05 | 0.27 | 0.23 | 1.25 | 5.3 | 0.045 | 10.25 | 0.107 |
| ***TNC*** | Bone | 1.67 | 8.74 | 3.3 | 15.29 | 5.2 | 0.064 | 4.331 | 0.088 |

*BGN*, biglycan; *COL1A1*, type I collagen, alpha 1 chain; *COL2A1*, type II collagen alpha 1 chain; *COL3A1*, type III collagen alpha 1 chain; *CSPG2*, chondroitin sulphate proteoglycan 2; *DCN*, decorin; *LUM*, lumican; *MMP13*, matrix metalloproteinase 13; *TIMP1*, tissue inhibitor of metalloproteinase 1; *TIMP2*, tissue inhibitor of metalloproteinase 2; *TIMP4*, tissue inhibitor of metalloproteinase 4; *TNC*, Tenascin C
