# Supplementary material for: Histological and molecular characterisation of feline humeral condylar osteoarthritis
Source: BMC Vet Res. 2013 Jun 4;9:110. doi: 10.1186/1746-6148-9-110 (PMC3681712; doi:10.1186/1746-6148-9-110)
Supplement: Additional file 3 — Primer and probe sequences. [file 1746-6148-9-110-S3.docx]

**Table 3**

Primer and probe sequences

| **Gene** | **Ensembl Transcript Number / Accession number** | **Forward Primer** | **Exon Number** | **Reverse Primer** | **Exon Number** | **Probe** | **Exon Number** | **Amplicon Length** | **Amplicon Sequence** |
| --- | --- | --- | --- | --- | --- | --- | --- | --- | --- |
| ***ATIC*** | ENSFCAT00000009218 | GGAACCATTGGCGAGGAT | 15-16 | CTGCTTCAGTTAGCAGTTCAGG | 16 | TGGAAGGC | 16 | 79 | ggaaccattggcgaggatgaagatttggtaaagtggaaggcattgtttgaggaagtccctgaactgctaactgaagcag |
| ***BGN*** | ENSFCAT00000004030 | CGAGAACGGGAGCCTAAGTT | 6 | TGGTGTGCAGATAGACCACCT | 7 | GGGAGCTG | 6-7 | 125 | cgagaacgggagcctaagttttctgcccaccctgcgggagctgcacttggacaataacaagctgtccagggtgccttctggccttccggacctcaagctcctccaggtggtctatctgcacacca |
| ***COL1A1*** | ENSFCAT00000004567 / AC236832.1 | AGGTCCTTCCGGAGCTTCT | 46 | ACCGTTGAGTCCGTCCTTC | 47 | TCCTGCTG | 47 | 85 | aggtccttccggagcttctggtcctgctggtccccgaggtccccccggcgctgctggctctcctgggaaggacggactcaacggt |
| ***COL2A1*** | ENSFCAT00000014006 | AGCGAGTGTTCCCAAGAAGA | 52 | GGTTGTCATCTCCATAGCTGAAG | 52-53 | TGGTGGAG | 52 | 113 | agcgagtgttcccaagaagaactggtggagcagcaagagcaaggacaagaagcacatctggttcggagaaaccatcaacggtggcttccacttcagctatggagatgacaacc |
| ***COL3A1*** | ENSFCAT00000014744 | CTGGCCAGCCTGGAGATA | 36 | CCATTCTGTCCAGGAGCAC | 38-39 | ACCTGCTG | 38 | 139 | ctggccagcctggagataagnnnnnnnnnnnnnnnnnnnnnnnnnnnnnnnnnnnnnnnnnnnnnnnnnnnnnnggtgaaagaggtgaacatgggcctccaggacctgctggcttccctggtgctcctggacagaatgg |
| ***CSPG2*** | ENSFCAT00000009211 | GCAGCACACTGCAATATGAGA | 20-21 | TCTTCTCCAGCAGAAAAGAAGC | 21 | CCAGCCAG | 21 | 67 | gcagcacactgcaatatgagaactggaggcccaaccagccagacagcttcttttctgctggagaaga |
| ***DCN*** | ENSFCAT00000000751 | TCAAGAACCTGAAGAACCTTCATA | 2-3 | GTTCCAATTTCAACAAAGGTGTAA | 3 | CCTGGAGC | 3 | 96 | tcaagaacctgaagaaccttcatacgttgatccttgtcaacaacaaaattagcaaaatcagccctggagcatttacacctttgttgaaattggaac |
| ***GAPDH*** | ENSFCAT00000006876 / NM_001009307.1 | TGGAGTCTACTGGGGTCTTCA | 2 | CAGAAGGGGCAGAGATGATG | 3 | CTGGGGCT | 2-3 | 90 | tggagtctactggggtcttcaccaccatggagaaggctggggctcacttgaagggtggggccaagagggtcatcatctctgccccttctg |
| ***LUM*** | ENSFCAT00000003763 | ACTCCCCAAGTCCCTGGT | 1 | GAGCCAAGCTTCGTTATCTTGT | 1 | CTCCAGCT | 1 | 60 | actccccaagtccctggtggatctccagcttacgcacaacaagataacgaagcttggctc |
| ***MMP13*** | ENSFCAT00000000118 | CTCTTCTTCTCGGGAAACCA | 8 | GGGGGAGTCCTGATCCAT | 9 | TCTGGAGC | 8-9 | 62 | ctcttcttctcgggaaaccaggtctggagctatgatgacagcaaccacgccatggatcaggactccccc |
| ***MRPS25*** | ENSFCAT00000003665 | GAGATCATGGAGCACGTCAA | 5 | CCTCTCTCTCCAGGGTTTCC | 6 | TGGGGAAG | 5 | 61 | gagatcatggagcacgtcaaaaaaatcctggggaagagcaaggaaaccctggagagagagg |
| ***MRPS7*** | ENSFCAT00000014520 | ATGGCGCAGACTCTGGAA | 3-4 | GCAGAGGCAGCATGGTACT | 4 | GGAAGCAG | 4 | 62 | atggcgcagactctggaagctgtgaaaaggaagcagtttgagaagtaccatgctgcctctgc |
| ***TIMP1*** | ENSFCAT00000008192 | TGCGAAGAATGCACCGTA | 3-4 | AAACCCTTGTCAGTGCCTGT | 4 | CCAGTTCC | 4 | 107 | tgcgaagaatgcaccgtattttcctgttcatccatcccctgcaaactgcagaatgacactcactgcttgtggacagaccagttcctcacaggcactgacaagggttt |
| ***TIMP2*** | ENSFCAT00000002548 /AY250765.1 | ATGGGCTGTGAGTGCAAGAT | 4-5 | GTCCGGAGACGAGATGTAGC | 5 | CTGCCCCA | 5 | 63 | atgggctgtgagtgcaagatcacgcgctgccccatgatcccgtgctacatctcgtctccggac |
| ***TIMP4*** | ENSFCAT00000006232 | TCTCTCTGTGGTGTAAAACTAGAAGC | 4 | TCCATCACTGAGGATCTGACC | 4-5 | AGCCAGAA | 4 | 75 | tctctctgtggtgtaaaactagaagctaacagccagaagcaatatctcttgactggtcagatcctcagtgatgga |
| ***TNC*** | ENSFCAT00000004942 | CTATCGCAACTGGAAAGCCTA | 25 | GTAGATTGTCCAGCCCAAGC | 25-26 | CTGCTGGG | 25 | 77 | ctatcgcaactggaaagcctacgctgctgggtttggggaccgcagagaagaattctggctTgggctggacaatctac |

*ATIC* 5-aminoimidazole-4-carboxamide ribonucleotide formyltransferase/IMP cyclohydrolase; *BGN*, biglycan; *COL1A1*, type I collagen, alpha 1 chain; *COL2A1*, type II collagen alpha 1 chain; *COL3A1*, type III collagen alpha 1 chain; *CSPG2*, chondroitin sulphate proteoglycan 2, or versican; *DCN*, decorin; *GAPDH*, glyceraldehyde-3-phosphate dehydrogenase; *LUM*, lumican; *MMP13*, matrix metalloproteinase 13; *MRP S7,* mitochondrial ribosomal protein S7; *MRP S25,* mitochondrial ribosomal protein S25; *TIMP1*, tissue inhibitor of metalloproteinase 1; *TIMP2*, tissue inhibitor of metalloproteinase 2; *TIMP4*, tissue inhibitor of metalloproteinase 4; *TNC*, Tenascin C
