# Supplementary material for: Histological and molecular characterisation of feline humeral condylar osteoarthritis
Source: BMC Vet Res. 2013 Jun 4;9:110. doi: 10.1186/1746-6148-9-110 (PMC3681712; doi:10.1186/1746-6148-9-110)
Supplement: Additional file 4 — MiQE checklist. [file 1746-6148-9-110-S4.docx]

**Additional files**

**Additional file 1**

| **Sample/Template** | **Details** | **Checklist** |
| --- | --- | --- |
| Source | If cancer, was biopsy screened for adjacent normal tissue? | Ostearthritic tissues, conifmred by histology and radiography |
| Method of preservation | Liquid N2; RNAlater; formalin | RNAlater |
| Storage time (if appropriate) | If using samples >6 months old | <6 months |
| Handling | fresh; frozen; formalin | Fresh |
| Extraction method | TriZol; columns | Trizol/Chloroform and Rneasy columns |
| RNA: DNA-free | Intron-spanning primers; no RT control | Dnase on-column treatment, intron spanning primers where possible, genomic contamination RT/PCR check |
| Concentration | Nanodrop; Ribogreen; microfluidics | Nanodrop |
| RNA: integrity | Microfluidics; 3':5' assay | RIN (Ref 48) |
| Inhibition-free | Method of testing | Not done |
| **Assay optimisation/validation** |  |  |
| Accession number | RefSeq XX_1234567 | Table 1 |
| Amplicon details | exon location, amplicon size, amplicon sequence | Table 1 |
| Primer (and if used probe) sequence | even if previously published | Table 1 |
| amplicon verification | melt-curves | Probes used, Agarose gel confirmation of amplicon size |
| *In silico* | BLAST; Primer-BLAST; m-fold | BLAST |
| empirical | primer concentration; annealing temperature | Concentration standardised, in Material and Methods section |
| Priming conditions | oligo-dT; random; combination; target-specific | Oligo dT |
| PCR efficiency | dilution curve | >93% <107% |
| Linear dynamic range | spanning unknown targets | 1-1000 dilution |
| Limits of detection | LOD detection; accurate quantification | Not done |
| Intra-assay variation | copy numbers not Cq | Not done |
| **RT/PCR** |  |  |
| Protocols | detailed description, concentrations, volumes | Described in Materials and Methods section |
| Reagents | supplier, Lot number | Described in Materials and Methods section |
| Duplicate RT | DCq | Single RT |
| NTC (no template controls) | Cq & melt curves | Included |
| NAC (no amplification controls) | DCq beginning:end of qPCR (only for probe-based reactions) | Not done |
| Positive control | inter-run calibrators | Not done |
| **Data analysis** |  |  |
| Statistical justification | e.g., biological replicates | Duplicate |
| Transparent, validated normalisation | e.g., GeNorm summary | GeNorm |
| Specialist software | e.g., QBAsePlus | Not required |
